# Loss of the N-terminal domain of chlorophyllide *a *oxygenase induces photodamage during greening of Arabidopsis seedlings

**DOI:** 10.1186/1471-2229-8-64

**Published:** 2008-06-12

**Authors:** Akihiro Yamasato, Ryouichi Tanaka, Ayumi Tanaka

**Affiliations:** 1 Institute of Low Temperature Science, Hokkaido University, Sapporo 060-0819, Japan; 2 Division of Photobiology, National Institute for Basic Biology, Okazaki 444-8585, Japan

## Abstract

**Background:**

Chlorophyll *b *is a major photosynthetic pigment in green plants that is synthesized by chlorophyllide *a *oxygenase (CAO). The regulation of chlorophyll *b *biosynthesis is an important determinant for the antenna size of photosystems. Chlorophyll *b *synthesis is partly regulated on a transcriptional level by the expression of the *CAO *gene. In addition, the synthesis of chlorophyll *b *is strictly regulated on a protein level by the stability of the CAO enzyme. CAO consists of three domains, which are sequentially named from the N terminus as the A, B and C domains. The A domain of CAO participates in the regulation of the CAO protein stability.

**Results:**

In order to clarify the physiological function of the A domain, we constructed transgenic Arabidopsis (*Arabidopsis thaliana*) plants which either overexpressed the complete CAO or a truncated version of CAO lacking the A domain. The transgenic plants overexpressing the A-domain-deleted CAO accumulated an excess amount of chlorophyll *b *during greening. The transgenic plants which lacked the A domain either died or were obviously retarded when they were exposed to continuous light immediately after etiolation. In addition, the loss of the A domain in CAO impaired another step of chlorophyll biosynthesis, namely the conversion of divinyl-protochlorophyllide *a *to monovinyl protochlorophyllide *a *under dark conditions.

**Conclusion:**

The A domain of CAO regulates the level of CAO, and thus prevents the excess accumulation of chlorophyll *b*. This function of the A domain is especially important during the greening stage of etiolated seedlings. At this stage, the plants are vulnerable to photodamages which could be caused by excessive chlorophyll *b *accumulation. In addition, de-regulation of the CAO level affects monovinyl-protochlorophyllide biosynthesis in darkness by unknown mechanisms. In conclusion, the A domain of CAO is essential in the control of chlorophyll biosynthesis and in the survival of seedlings during de-etiolation especially under strong illumination.

## Background

Chlorophyll molecules are major components of photosynthesis and play essential roles in harvesting light energy and charge separation. Chlorophylls are actively synthesized during greening and are assembled with various apoproteins to form chlorophyll-protein complexes. Apoproteins of core antenna complexes solely bind chlorophyll *a*, while apoproteins of peripheral antenna complexes bind both chlorophyll *a *and chlorophyll *b *[1]. Since these chlorophyll-protein complexes are stoichiometrically assembled to form functional photosystems, chlorophyll metabolism must be tightly regulated to coordinate with the formation of these complexes. Previous reports have suggested that the regulation of chlorophyll metabolisms is important from the viewpoint of photodamage. Most of the chlorophyll intermediates are photoreactive compounds [2]. If the regulation of 5-aminolevulinic acid (ALA), a precursor of tetrapyrrole synthesis, is defective as the result of mutation of a regulation factor, protochlorophyllide is accumulated in excessive amounts under dark conditions [3]. Consequently, this overabundance of protochlorophyllide results in the generation of reactive oxygen species (ROS) and finally causes growth retardation or cell death during greening [3].

At the last step of chlorophyll biosynthesis, chlorophyll *b *is synthesized from chlorophyll *a *by chlorophyllide *a *oxygenase (CAO)[4]. Since an increase in *CAO *mRNA levels stimulates chlorophyll *b *synthesis, CAO activity is considered to be partly regulated by *CAO *gene expression [5-7]. In addition to the regulation on the transcriptional level, chlorophyll *b *synthesis is also regulated by protein stability. CAO consists of three domains, which are named sequentially from the N terminus as the A, B and C domains [8]. The C domain contains binding motifs for a Rieske center and non-heme iron and catalyzes the conversion of chlorophyll *a *to chlorophyll *b *[4,8,9]. Although the precise function of the B domain is not known, it is considered to possibly function as a linker between the A and C domains [10]. Although the A domain is not involved in catalytic function, it regulates CAO protein stability [8,11]. When the A domain was removed from CAO, and only BC domains were introduced and overexpressed in Arabidopsis (*Arabidopsis thaliana*), the protein levels of CAO were drastically increased and chlorophyll *b *was excessively accumulated [11]. We recently determined that the chloroplast Clp protease plays an important role in CAO protein stability [12]. However, excess chlorophyll *b *accumulation does not result in a distinct alteration of photosynthetic activity and does not induce photodamage in green leaves [13]. Therefore, at the present time it is still not known why the protein level of CAO is strictly regulated by the A domain.

In order to answer this question, we introduced genes corresponding to either the complete CAO sequence or BC domains into Arabidopsis. We subsequently investigated the greening processes of these transgenic plants under various light conditions. Interestingly, transgenic plants expressing only the BC domains died when the etiolated seedlings were exposed to continuous light. In addition, chlorophyll synthesis was disturbed in these plants during etiolation and greening of seedlings. We discuss the role of the A domain in the regulation of chlorophyll synthesis and chloroplast development.

## Results

### CAO without the A domain caused photodamage during greening

**Figure 1:**
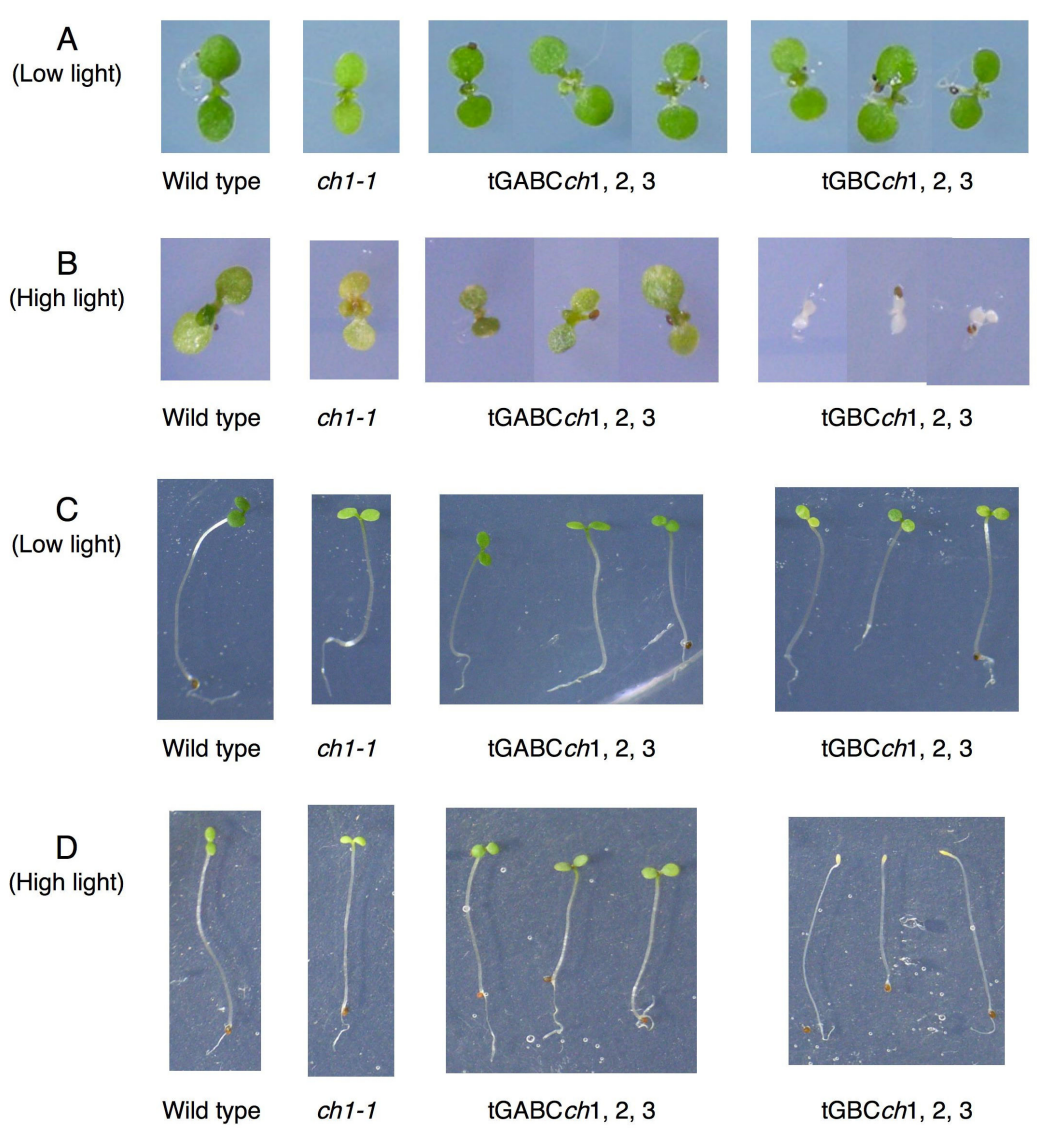
**Light sensitivities of the Arabidopsis seedlings**. A and B: Arabidopsis seedlings were germinated and grown continuously under low (70 μE m^-2 ^s^-1^) or high light (300 μE m^-2 ^s^-1^) conditions for 7 days. C and D: Etiolated seedlings were grown for 4 days and transferred to low or high light conditions for an additional 3 days. Numbers after transgenic plants (for example, tGBC*ch*1, 2, 3) indicate independent transgenic lines for each type of transgenic plants.

In order to elucidate the physiological role of the A domain, we examined the light sensitivity of two types of transgenic plants that we named tGBC*ch *and tGABC*ch *in the *ch1-1 *mutant background. The *ch1-1 *mutant contains a deletion in the *CAO *gene and does not contain chlorophyll *b *[14]. The tGBC*ch *lines overexpress a chimeric transgene containing the *GFP *gene and the *CAO *gene lacking the A-domain coding sequence [11]. In contrast, the tGABC*ch *lines overexpress another chimeric transgene encoding GFP and the full-length CAO protein [11]. Both types of transgenic plants were grown under different light conditions and their phenotypes were compared to those of wild type and the *ch1-1 *mutant. When the seeds were germinated and grown under low light conditions, all four strains grew well, although the *ch1-1 *exhibited a pale green phenotype (Fig. 1A). When etiolated seedlings were transferred to low light conditions, most of the tGBC*ch *plants were bleached and the growth of the tGBC*ch *seedlings was retarded (Fig. 1C). When exposed to high light conditions, no etiolated and green seedlings of tGBC*ch *survived (Fig. 1B and 1D). On the contrary, seedlings from the other three strains developed normally and grew under low and high light conditions (Fig. 1B, C and 1D). These results confirmed that the overexpression of the *CAO *gene lacking the A domain resulted in photodamage to Arabidopsis seedlings.

**Figure 2:**
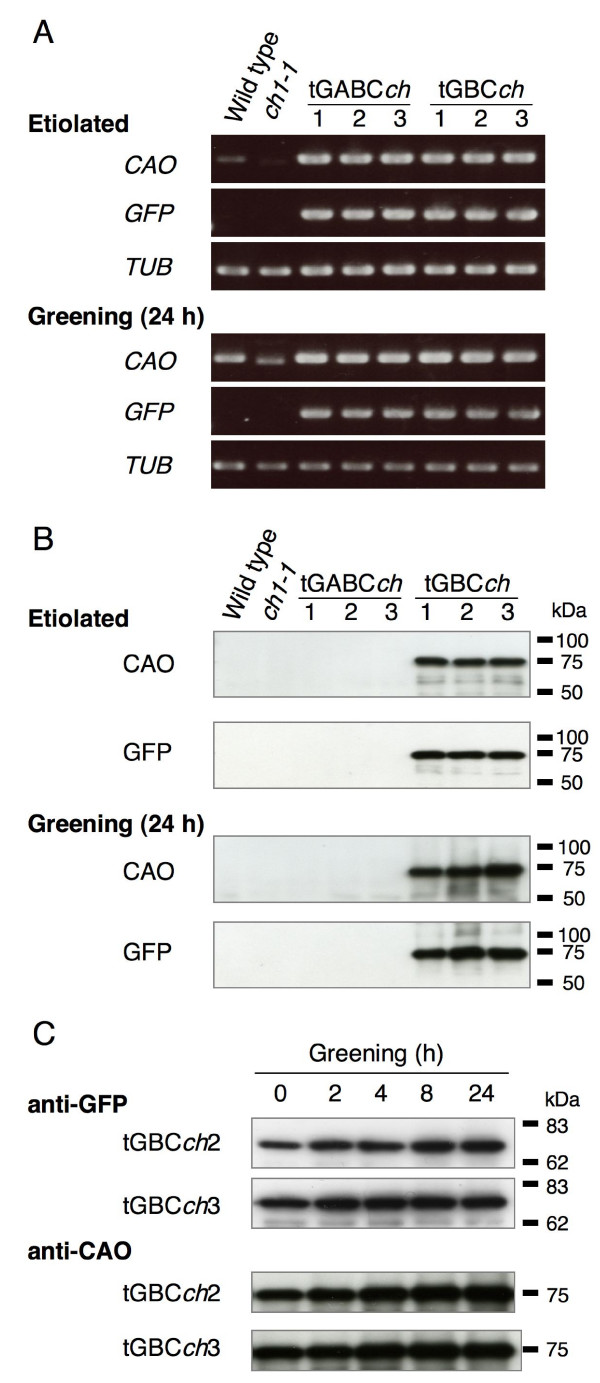
**Expression levels of CAO in the etiolated and greening seedlings**. A: The expression levels of internal *CAO *gene and *GFP-CAO *transgenes in 4-day-old etiolated seedlings and greening seedlings were determined by semi-quantitative PCR. Expression levels from the *tubulin *(*TUB2*) gene were monitored as a quantitative control. The internal *CAO *expression level increased in wild type and *ch1-1 *seedlings during greening. The *CAO *transcript of *ch1-1 *has a 31 bp deletion and it was not detected in the tGABC*ch *and tGBC*ch *plants. B: The accumulation levels of the CAO proteins and GFP-CAO fusion proteins in 4-day-old etiolated seedlings and in greening seedlings were determined. The estimated molecular size of the full-length CAO, GFP-ABC and GFP-BC proteins without transit peptide were 56, 83 and 70 kDa, C: The accumulation levels of the GFP-BC proteins in 4-day-old etiolated tGBC*ch *seedlings during a 24 h greening period were determined. The full-length CAO or GFP-CAO fusion proteins were not detected in wild type, *ch1-1 *and tGABC*ch *seedlings (data not shown).

**Figure 3:**
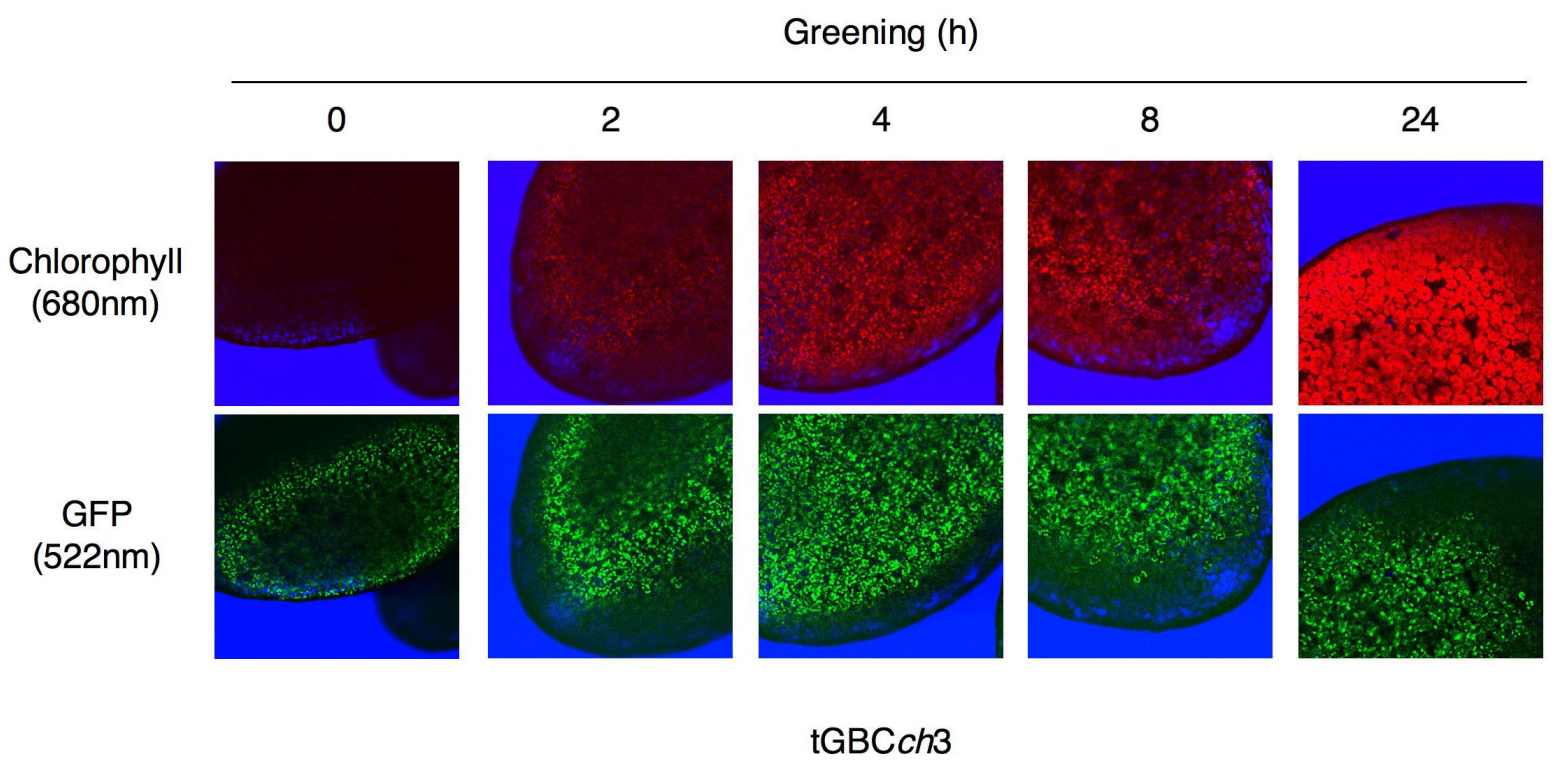
**Accumulation of chlorophyll and the GFP-BC protein in the tGBC*ch *seedlings during greening**. The fluorescence images of the Arabidopsis cotyledons during greening for 24 h were observed with confocal microscopy. The GFP-CAO protein and the plastids during greening are indicated by green and red fluorescence, respectively. The green fluorescence of GFP-BC protein was detected only within plastids during greening.

*GFP-CAO *fusion transgenes were highly overexpressed in the etiolated and greening seedlings of all transgenic tGABC*ch *and tGBC*ch *plant lines (Fig. 2A). The expression levels of the transgenes, which correspond to GFP-ABC or GFP-BC, were higher than that of full-length CAO in wild type seedlings (Fig. 2A). The tGBC*ch *transgenic lines accumulated a large amount of GFP-BC protein (a fusion of GFP and B-C domains of CAO) in both etiolated and green seedlings (Fig. 2B and 2C). The GFP-BC proteins localized to etioplasts and chloroplasts (Fig. 3). In contrast, the full-length CAO (A-B-C domains) and the GFP-full-length CAO (a fusion of GFP and A-B-C domains of CAO) proteins were under a detectable level by immunoblotting in both etiolated and greening seedlings (Fig. 2B). It was evident that the full-length CAO and the GFP-full-length CAO proteins accumulated to certain levels in the green leaves because chlorophyll *b *was actively synthesized in the transgenic plants [11]. However, the protein levels of CAO in the seedlings were too low for detection by immunoblot analysis. These data suggest that the low accumulation level of CAO is sufficient to synthesize chlorophyll *b*. We should note that the excess accumulation of the fusion protein in the GFP-BC overexpressing plants was not due to the transcriptional or translational activation in these plants [11]. It was most likely that the lack of the A domain impaired the degradation of CAO by Clp protease [12].

As shown in Figs. 1 and 2, our transgenic lines did not show any obvious phenotypical differences between the lines. In the subsequent experiments described within this study, we also examined all transgenic lines shown in Figs. 1 and 2. All of the lines produced results that were nearly identical.

**Figure 4:**
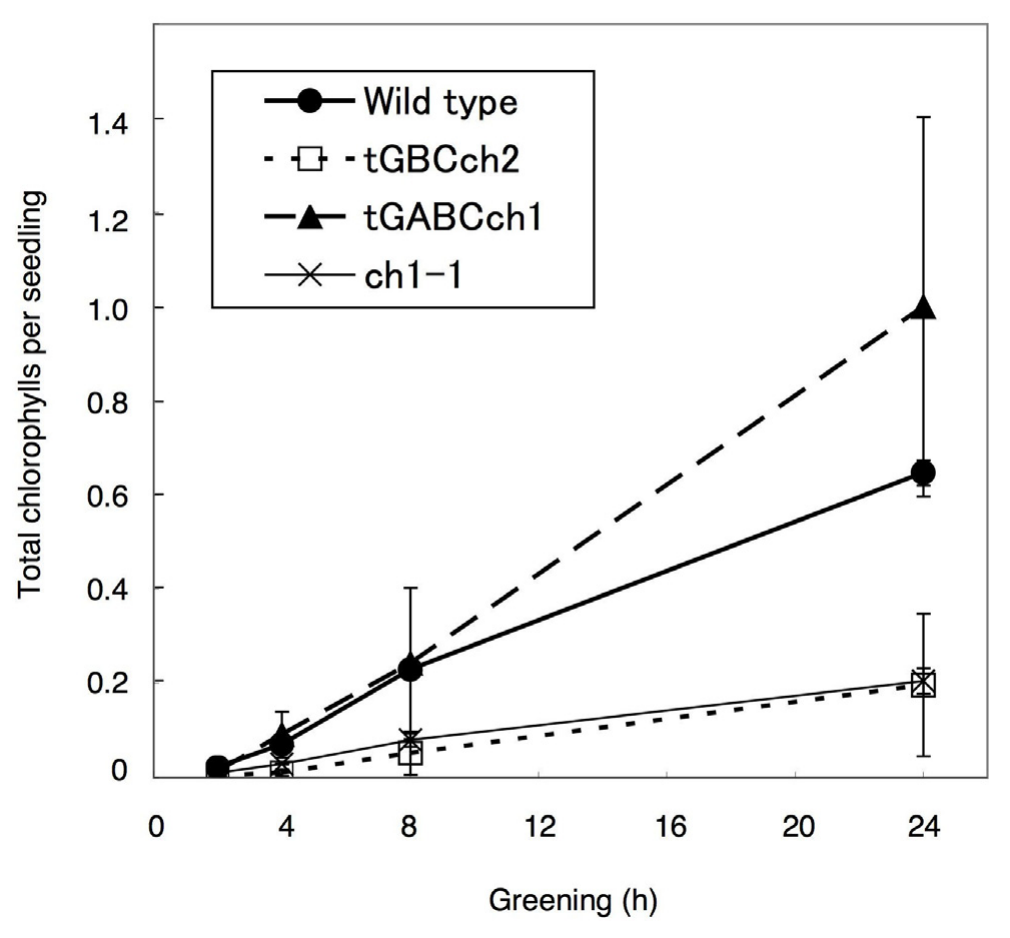
**Chlorophyll levels in the seedlings during greening**. Etiolated seedlings were grown for 4 days under dark conditions. The etiolated seedlings were then exposed to low light conditions and were harvested during greening at the indicated times. The total amount of chlorophyll per seedling was determined with HPLC analysis. The error bars represent standard deviations (*n *= 4).

**Figure 5:**
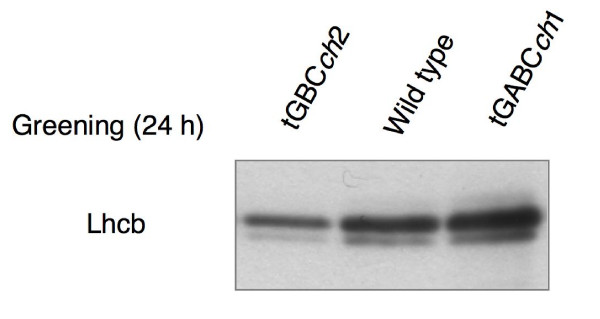
**Lhcb protein levels in the greening seedlings**. Etiolated seedlings were first grown for 4 days under dark conditions and were subsequently exposed to low light conditions for 24 h. The accumulation levels of the Lhcb proteins in the greening seedling were determined by immunoblotting using anti-Lhcb antibody. Lhcb protein levels in the *ch1-1 *seedlings after 24 h of greening were nearly equivalent to those from tGBC*ch *(data not shown).

**Figure 6:**
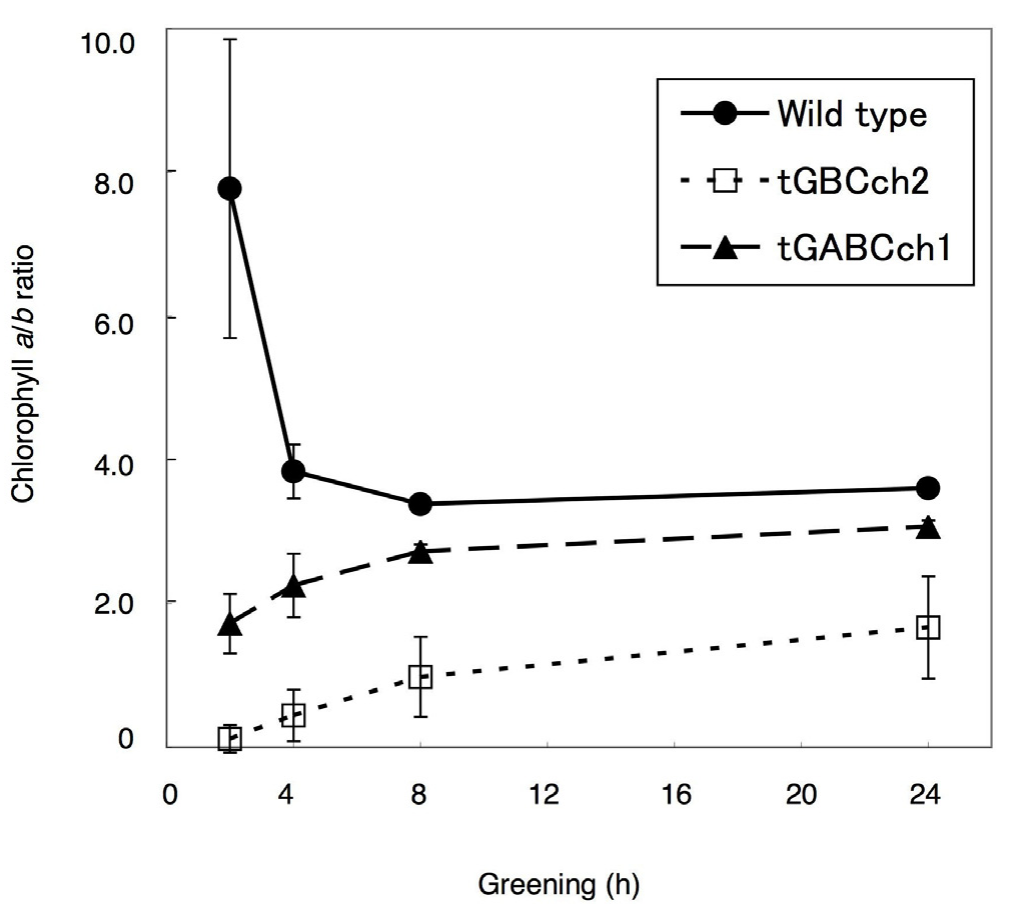
**Molar ratio of the chlorophyll *a *and *b *contents in the seedlings during greening**. The etiolated seedlings were first grown for 4 days under dark conditions and were subsequently exposed to low light. The compositions of chlorophyll *a *and *b *were calculated from chromatographic peak profiles at 650 nm with HPLC analysis. The error bars represent standard deviations (*n *= 4).

**Figure 7:**
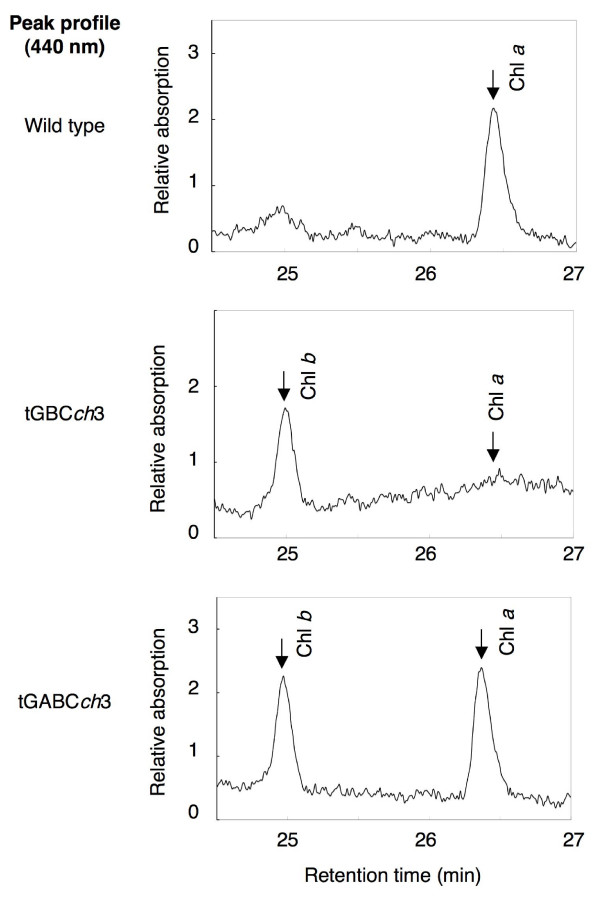
**Chlorophyll accumulation in etiolated Arabidopsis seedlings after light treatments**. Chlorophyll accumulation after exposure to low light for 5 min in 6-day-old etiolated seedlings. The chlorophyll *a *and *b *extracted from the seedlings were separated by HPLC analysis. Chl *a*, chlorophyll *a*; Chl *b*, chlorophyll *b*.

Next, we examined the accumulation of chlorophyll when etiolated seedlings were transferred to continuous light conditions. In comparison to wild type, chlorophyll levels were low in tGBC*ch *and *ch1-1 *but slightly higher in tGABC*ch *(Fig. 4). In good accordance with chlorophyll accumulation, Lhcb protein levels were higher in tGABC*ch *and lower in tGBC*ch *compared to wild type (Fig. 5). The chlorophyll *a/b *ratio was very high soon after the onset of illumination and decreased gradually during the greening in wild type (Fig. 6). In contrast, the chlorophyll *a/b *ratio was already low after illumination and gradually increased during greening in tGABC*ch *and tGBC*ch *(Fig. 6). In tGBC*ch*, the chlorophyll *a/b *ratio was extremely low. Fig. 7 shows the HPLC profiles of chlorophyll that accumulated after 5 min of illumination in wild type and two types of transgenic plants. Under this condition, most of the chlorophyll molecules were derived from protochlorophyllide *a *that pre-accumulated during the dark condition. The wild type seedlings accumulated chlorophyll *a *but not chlorophyll *b *(Fig. 7). It should be noted that the small peak at 25 min in wild type was not chlorophyll *b *because the spectrum showed different peaks (data not shown). In contrast, the tGBC*ch *only accumulated chlorophyll *b*, suggesting that chlorophyll *a *(or chlorophyllide *a*) in tGBC*ch *was immediately converted to chlorophyll *b *(or chlorophyllide *b*) after the photoconversion of protochlorophyllide *a *into chlorophyllide *a *(Fig. 7). Both chlorophyll *a *and chlorophyll *b *accumulated in tGABC*ch *and the level of chlorophyll *a *was nearly equivalent to the levels of wild type (Fig. 7).

**Figure 8:**
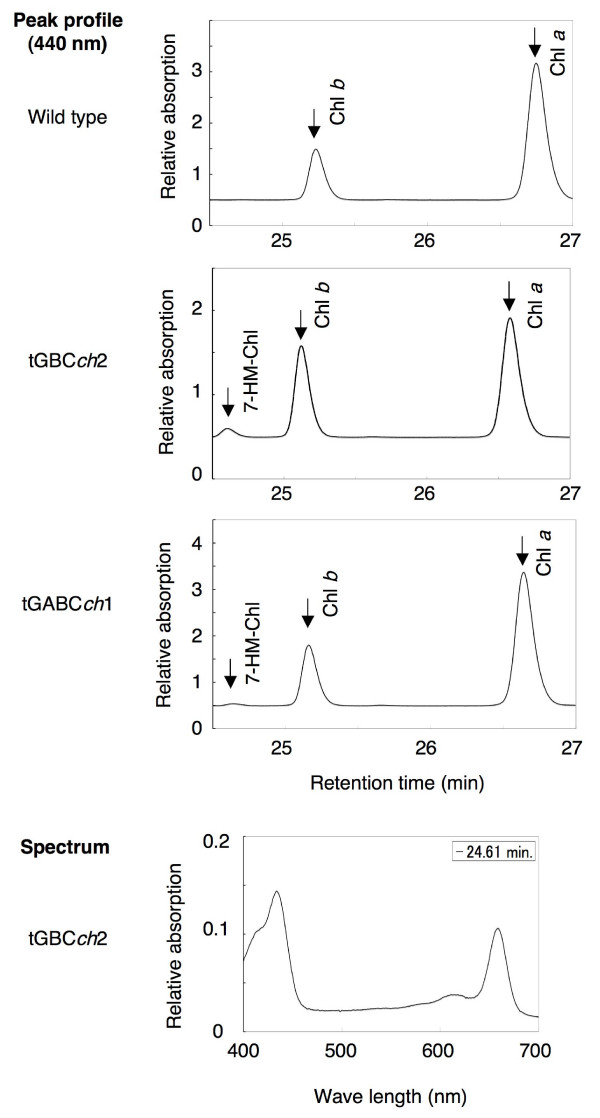
**Chlorophyll accumulation in greened seedlings**. Chlorophyll accumulation in fully greened seedlings that were grown for 8 days under low light conditions. The chlorophyll *a*, *b *and 7-hydroxymethyl chlorophyll extracted from the seedlings were separated by HPLC analysis. The spectrum of a peak that was eluted at 24.6 min in the extract from the tGBC*ch *leaves is shown. Chl *a*, chlorophyll *a*; Chl *b*, chlorophyll *b*; 7-HM-Chl, 7-hydroxymethyl chlorophyll.

Interestingly, we found that the fully-greened leaves of tGBC*ch *accumulated a significant amount of 7-hydroxymethyl chlorophyll, which is an intermediate molecule in the conversion between chlorophyll *b *and chlorophyll *a *(Fig. 8). The unusually high level of 7-hydroxymethyl chlorophyll suggests that the excess GFP-BC proteins or the excess chlorophyll *b *might activate the conversion between chlorophyll *a *and *b *[15].

### Loss of the A domain results in a perturbation of chlorophyll metabolism

The tGBC*ch *seedlings accumulated a low level of chlorophyll during the greening process (Fig. 4). These data are consistent with the observation that the amount of protochlorophyllide, which is an end product of chlorophyll synthesis in etiolated seedlings, decreased in tGBC*ch *transgenic plants (Table 1). When plants were fed with ALA, similar trends were observed between wild type and the transgenic plants (Table 1). These results might suggest that certain enzymatic steps between ALA and protochlorophyllide formation were disturbed and that this disturbance limited the chlorophyll synthesis in the tGBC*ch *seedlings during greening.

**Table 1:** Total protochlorophyllide accumulation in etiolated seedlings.

Strains	Total protochlorophyllide/etiolated seedling (pmole)		Divinyl-protochlorophyllide/total protochlorophyllide (molar ratio)
		
	-ALA	+ALA	-ALA
Wild type	0.78 ± 0.02	8.09 ± 1.98	0.37 ± 0.03
tGBC*ch*3	0.33 ± 0.01	2.56 ± 0.56	0.80 ± 0.05
tGABC*ch*3	1.01 ± 0.09	6.29 ± 2.08	0.32 ± 0.03

The seedlings were grown for 6 days under dark conditions with or without ALA (0.5 mg ml^-1^) on wet filter paper. Seedlings were subsequently harvested under dark conditions and their extracts were analyzed for their protochlorophyllide composition by HPLC analysis (*n *= 5).

In our next line of investigation, we examined the re-accumulation of protochlorophyllide in the dark after a brief illumination as a means to analyze the kinetics of protochlorophyllide synthesis in the seedlings. Under these conditions, protochlorophyllide levels were similar in wild type, tGBC*ch *and tGABC*ch *during the first day of darkness (Table 2). However, the levels decreased in the following 2-day period of darkness only in tGBC*ch *(Table 2). Although the etiolated tGBC*ch *seedlings seemed to synthesize protochlorophyllide normally, the pigment appeared to be broken down during dark condition by unknown mechanisms. Alternatively, It is also possible that divinyl-protochlorophyllide *a *may inhibit an earlier step of chlorophyll biosynthesis in a feedback manner.

**Table 2:** Total protochlorophyllide accumulation in etiolated seedlings during dark incubation.

Strains	Total protochlorophyllide/etiolated seedling (pmole)		
	
	Dark incubation after light treatment		
	0 day	1 day	3 days
Wild type	n.d.	0.70 ± 0.01	0.77 ± 0.16
tGBC*ch*3	n.d.	0.49 ± 0.01	0.25 ± 0.02
tGABC*ch*3	n.d.	0.68 ± 0.13	1.28 ± 0.01

Six-day-old etiolated seedlings were exposed to low light for 5 min and were returned to dark conditions. The seedlings after a 1 or 3 day additional dark incubation period were subsequently harvested under dark conditions. Their extracts were analyzed for their protochlorophyllide composition by HPLC analysis (*n *= 3). The n.d. abbreviation indicates "not detected".

**Figure 9:**
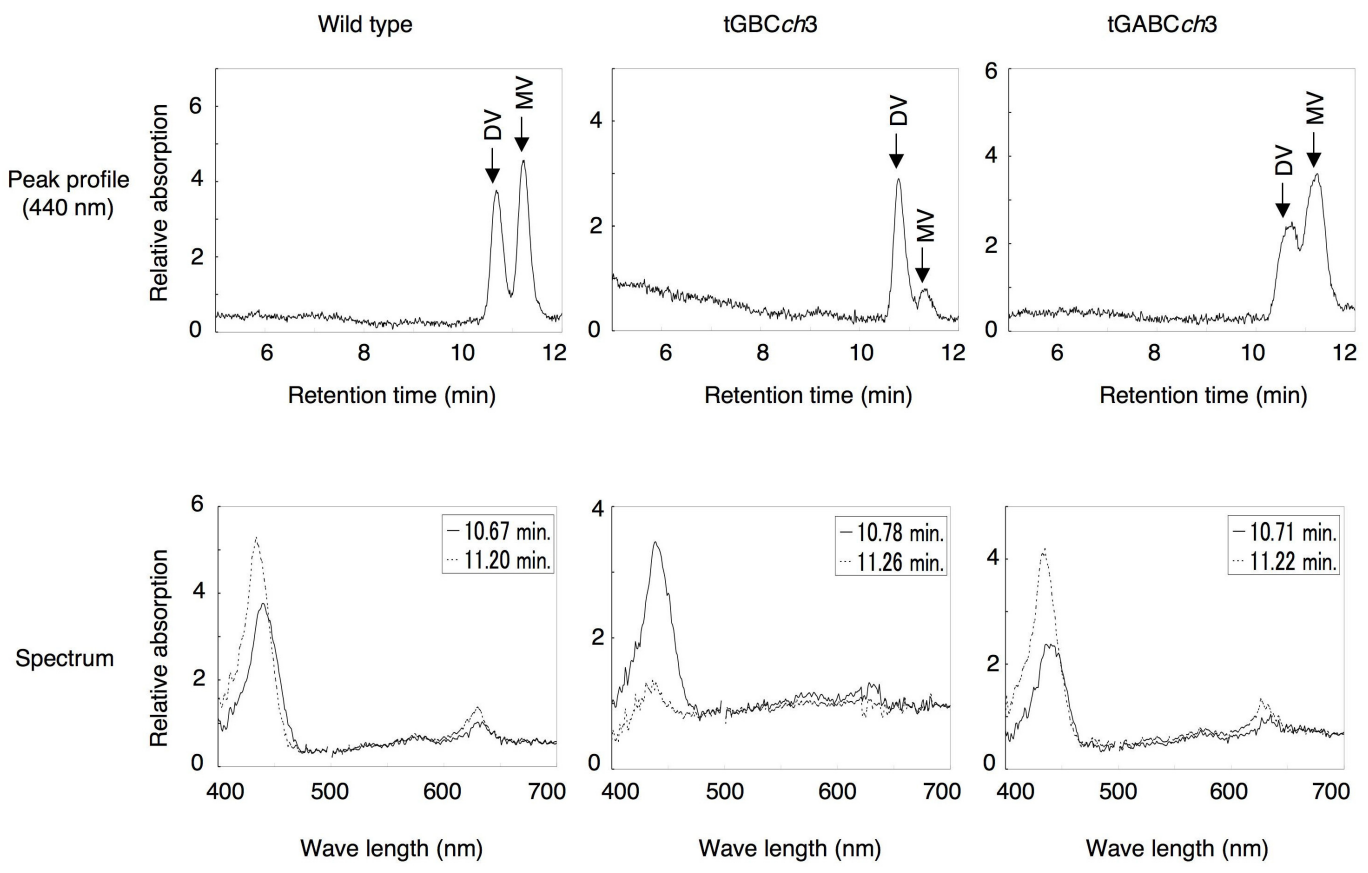
**Chromatographic peaks and spectra of divinyl- and monovinyl-protochlorophyllide *a *in etiolated seedlings**. Seedlings were grown for 6 days under dark conditions on wet filter paper without ALA. The seedlings were harvested under dark conditions and their extracts were analyzed for their protochlorophyllide composition by HPLC. DV, divinyl-protochlorophyllide *a*; MV, monovinyl-protochlorophyllide *a*.

In order to investigate the chlorophyll *b *synthetic pathway in tGBC*ch*, we compared the compositions of the chlorophyll intermediates in wild type, tGBC*ch *and tGABC*ch*. In spite of the accumulation of CAO in tGBC*ch*, protochlorophyllide *b *was not detected in etiolated seedlings (Fig. 9). These data indicate that CAO is unable to catalyze the conversion of protochlorophyllide *a *to protochlorophyllide *b *in Arabidopsis. Interestingly, a significant difference was observed in the accumulation ratios of divinyl-protochlorophyllide *a *to total protochlorophyllide *a *among wild type, tGBC*ch *and tGABC*ch *(Fig. 9 and Table 1). The etiolated seedlings of wild type and tGABC*ch *predominately accumulated monovinyl-protochlorophyllide *a *(Table 1). Conversely, monovinyl-protochlorophyllide *a *was accumulated to very low levels in tGBC*ch *(Table 1). This observation indicates that the loss of the A domain from CAO resulted in an inactivation of 3, 8-divinyl protochlorophyllide *a *8-vinyl reductase [16].

## Discussion

### The A domain protects cells against photodamage during greening

We previously reported on the light sensitivity of transgenic Arabidopsis plants overexpressing prokaryotic CAO which does not have the A domain. These transgenic plants accumulated much more chlorophyll *b *than tGBC*ch *plants [13]. In this previous report, we concluded that the excess accumulation of chlorophyll *b *did not cause photodamage in the fully greened leaves of the transgenic plants [13]. However, in this study, we found that most of the etiolated tGBC*ch *seedlings died during greening not only under high light conditions, but also under low light conditions. Without the presence of chlorophyll *a*, chlorophyll *b *would not be incorporated into apoproteins. As a consequence, the free chlorophyll *b *might generate ROS [2]. In contrast to tGBC*ch*, transgenic plants overexpressing the full-length CAO accumulated both chlorophyll *a *and chlorophyll *b *immediately after the onset of illumination. Unlike tGBC*ch *plants, the full-length-CAO overexpression lines did not exhibit photodamage. It is possible that chlorophyll *b *is assembled with apoproteins in the presence of chlorophyll *a*. As a result, the generation of ROS might have been suppressed. Collectively, these results indicate that the A domain is essential in preventing photodamages that could be caused by excess chlorophyll *b *accumulation during greening. The etiolated seedlings of the transgenic Arabidopsis overexpressing prokaryotic CAO might cause obvious photodamage.

An unusually high level of 7-hydroxymethyl chlorophyll *a*, an intermediate molecule in the conversion between chlorophyll *b *to chlorophyll *a*, accumulated in the green leaves of tGBC*ch *(Fig. 8). This observation might indicate that excess chlorophyll *b *is reconverted to chlorophyll *a *by the chlorophyll cycle [15]. It is not likely that 7-hydroxymethyl chlorophyll was formed in the course of the chlorophyll *a *to chlorophyll *b *conversion. Since CAO catalyzes the two-step reaction from chlorophyll *a *to chlorophyll *b *by itself, the intermediate molecule, 7-hydroxymethyl chlorophyll, is supposed to be associated with the CAO enzyme and is not released during the reaction [14]. In our study, 7-hydroxymethyl chlorophyll was not found just after the start of the illumination of etiolated seedlings, although chlorophyll *b *was actively synthesized (Fig. 7). Therefore, it would be reasonable to assume that 7-hydroxymethyl chlorophyll was predominantly formed in the course of the chlorophyll *b *to chlorophyll *a *conversion.

### Accumulation of GFP-BC protein interferes with the regulation of chlorophyll biosynthesis

In comparison to wild type plants, chlorophyll accumulation during greening was suppressed in tGBC*ch *seedlings, even under low light conditions. It is possible that this suppression might be due to the oxidative stress generated by free chlorophyll *b*. The overabundance of protochlorophyllide generates ROS, which causes growth retardation or cell death during greening in the *flu *mutant [3]. Recently, we found that the chlorophyll biosynthesis pathway is a target of oxidative stress [17]. However, all of the phenotypes of tGBC*ch *could not be explained by oxidative stress alone. For example, the level of protochlorophyllide *a *in etiolated tissues of tGBC*ch *was lower than that of wild type. Since these plants were not exposed to light, the involvement of ROS in the inhibition of protochlorophyllide accumulation can be excluded. We also observed an increase in the ratio of divinyl-protochlorophyllide to the total protochlorophyllide in tGBC*ch *transgenic plants. These data indicate that the accumulation of the BC domains of CAO inhibits the activity of 3, 8-divinyl protochlorophyllide *a *8-vinyl reductase. On the contrary, overexpression of the GFP-ABC protein enhanced chlorophyll accumulation. These phenomena are very interesting in terms of biological regulation since both CAO and truncated CAO affected not only chlorophyll *b *synthesis but also other chlorophyll metabolic steps. These results are reasonable because some enzymes, which are related to chlorophyll biosynthesis, are reported to form complexes or interact with other enzymes. For example, glutamyl-tRNA reductase and glutamate-1-semialdehyde aminotransferase form a complex which enables an efficient substrate trafficking between two enzymes [18]. In addition, BchH, one of the three subunits of Mg chelatase of *Rhodobacter capsulatus*, accelerated the Mg-protoporphyrin IX monomethyl transferase activity [19]. If CAO does indeed interact with other enzymes, the accumulation of truncated CAO could potentially interfere with the activity of the target enzymes.

In addition, the loss of the A domain in CAO impaired the conversion of divinyl-protochlorophyllide *a *to monovinyl protochlorophyllide *a*, and obviously reduced total protochlorophyllide *a *level under dark conditions. A possible scenario for the reduction of protochlorophyllide accumulation in the tGBC*ch *seedlings is that the excessively-accumulated CAO in these seedlings interfered with the enzyme that catalyzes the formation of monovinyl-protochlorophyllide *a *from divinyl-protochlorophyllide *a*, the latter of which may inhibit an earlier step of chlorophyll biosynthesis in a feedback manner. Further studies are necessary and warranted to clarify this question.

### CAO does not catalyze the conversion of protochlorophyllide *a *to protochlorophyllide *b*

When etiolated seedlings of wild type are exposed to continuous light, chlorophyll *a *immediately accumulates after the onset of illumination. Subsequent to illumination, chlorophyll *a *and *b *levels gradually increase after several hours of a lag phase. Conversely, in tGBC*ch *plants, chlorophyll *b *accumulated but chlorophyll *a *was not detected after 5 min of illumination (Fig. 7). Considering that protochlorophyllide *b *was not found in etiolated seedlings, these data indicate that chlorophyll *a *was immediately converted to chlorophyll *b *in tGBC*ch *seedlings during the early phase of greening (Fig. 9).

Several research groups report that protochlorophyllide *b *does not exist in etioplasts [20,21]. Contrary to these reports, another group claimed that the protochlorophyllide *b *could be detected in etiolated seedlings when they use diethylpyrrocarbonate-containing acetone for the extraction of pigments [22]. It was also proposed that protochlorophyllide *b *is synthesized via a pathway that is independent from the CAO protein [23]. In addition, a cyanobacteria transformant with the *CAO *and *Lhcb *transgenes, accumulated protochlorophyllide *b *[24]. This observation indicates that CAO is capable of converting protochlorophyllide *a *to protochlorophyllide *b*. In this study, we used 100% acetone as an extraction solvent for chlorophyll intermediates in order to avoid the artificial formation of chlorophyll derivatives and to maximize our extraction of the chlorophyll intermediates from seedlings [21]. Consequently, we did not detect any protochlorophyllide *b *in etiolated tGBC*ch *seedlings, even though a large amount of the CAO protein accumulated. These results indicate that protochlorophyllide *b *is not a major intermediate product for chlorophyll *b *synthesis *in planta*.

## Conclusion

Taken together, our results demonstrated that excessive accumulation of chlorophyll *b *is harmful to plants. This deleterious effect likely disturbs the proper synthesis of chlorophyll intermediates and the accumulation of photosynthetic proteins. Therefore, we conclude that the regulation of the CAO protein by the A domain is essential for the survival of etiolated seedlings under light conditions. Since the A domain functions to maintain proper chlorophyll *b *levels throughout the development of plants [11], it would be reasonable to assume that the regulation of the CAO protein level is essential in various aspects of plant development and acclimation to light conditions. Further studies on the regulatory mechanism of CAO may elucidate the physiological roles of this mechanism throughout the life cycle of plants.

## Methods

### Plant materials and growth conditions

The Arabidopsis CAO protein consists of three domains, which are respectively named: A (V37-L170), B (P171-G200) and C (A201-G537) domains [8]. In this study, we used *Arabidopsis thaliana *Columbia wild type and the *ch1-1 *mutant which contains a deletion in the *CAO *gene [14]. In addition, we also used two types of transgenic plants overexpressing chimeric fusions of the CAO protein and the GFP. The two Arabidopsis transformants, tGBC*ch *and tGABC*ch*, expressed fusion transgenes corresponding to transit peptide-GFP-B-C domains and transit peptide-GFP-A-B-C domains in the *ch1-1 *background, respectively [11]. Three homozygous lines for each transgenic plant tGBC*ch *and tGABC*ch*, were used in this study. All of the transgenic plants were isolated independently. Arabidopsis seedlings were grown at 22°C on agar plates (0.7% [w/v]) containing 1/2 diluted Murashige-Skoog medium. Plants were grown under low light (70 μE m^-2 ^s^-1 ^from fluorescent bulbs) or high light (300 μE m^-2 ^s^-1 ^from xenon light bulbs [3.6 kW] equipped with ND filters, TGE-2S3, Tabai, Osaka, Japan) conditions.

### Reverse transcriptase PCR

Total RNA was extracted using an RNeasy Plant Mini Kit (Qiagen, Hilden, Germany) from 200 etiolated seedlings which were grown under dark conditions for 4 days. RNA was extracted from etiolated seedlings after a period of greening for 24 hours. One μg of isolated RNA was reverse-transcribed into cDNA using a SuperScript III Kit (Invitrogen, Carlsbad, CA). PCR was performed to determine gene expression level using the synthesized first-stranded cDNA sample in a 25 μl reaction. PCR conditions were as follows: 96°C (2 min); 25 cycles of 96°C (30 sec), 55°C (30 min), 72°C (1 min). The amount of cDNA template for the PCR was normalized by β-*tubulin *(*TUB2*) gene expression level. The primers used for RT-PCR were as follows: For *TUB2*, 5'-CTC AAG AGG TTC TCA GCA GTA-3' and 5'-TCA CCT TCT TCA TCC GCA GTT-3'; For *CAO*, 5'-AAC GAG GGA CGT ATT CAA TGT CCG-3' and 5'-AGA AGA AGG TAA ACA GAC ATG G-3'; For *GFP*, 5'-ATG GTG AGC AAG GGC GAG G-3' and 5'-TTA CTT GTA CAG CTC GTC CA-3'.

### Immunoblotting

Ten seedlings were homogenized with 100 μl of extraction buffer (50 mM Tris [pH 6.8], 2 mM EDTA, 10% [w/v] glycerol, 2% [w/v] SDS, 6% [v/v] 2-mercaptoethanol). The isolated supernatants (25 μl) were subsequently subjected to SDS-PAGE. The resolved proteins were blotted onto a hybond-P membrane (GE Healthcare, Buckinghamshire, UK). A 1/5000-diluted anti-GFP (Invitrogen) antibody, a 1/2000-diluted anti-CAO antibody [11] and a 1/5000-diluted anti-Lhcb [6] rabbit antibody were used to detect specific proteins as previously described [11]. Cross-reactive protein bands were developed using anti-rabbit IgG that was linked to horseradish peroxidase (GE Healthcare). An ECL plus western blotting analysis kit was use for the chemiluminescent detection of antigens (GE Healthcare).

### Confocal microscopy

Fluorescence images were recorded on an Axioplan fluorescence microscope (× 20 objective lens, Carl Zeiss, Jena, Germany) which was integrated in an MRC 1024 confocal laser-scanning microscopic system (Bio-rad laboratories, Hercules, CA). Samples were excited by an argon laser (25 mW) at 488 nm and the GFP and chlorophyll fluorescence were recorded at 522 and 680 nm, respectively. The images were processed with the Adobe Photoshop 4.0 software (Adobe, San Joze, CA).

### HPLC analysis

Chlorophylls were initially extracted from the seedlings with 100% acetone and the extracts were subsequently diluted to 80% acetone with water. Diluted extracts were then subjected to HPLC analysis (Shim-pack CLC-ODS column, 6.0 × 150 mm; Shimadzu, Kyoto, Japan) using methanol as the elution agent at a flow rate of 1.7 ml min^-1^. The compositions of chlorophyll species were calculated from chromatographic peak profiles at 650 nm [6].

Protochlorophyllides extracted from the seedlings were analyzed by HPLC on a Symmetry C8 column (4.6 × 150 mm; Waters Corporation, MA, USA) according to the previously described method of Zapata et al. [25]. Elution profiles and the spectra of the eluted pigments were recorded continuously in the range of 400 to 700 nm by SPD-M10A_AV _(Shimadzu). The compositions of protochlorophyllide were calculated from chromatographic peak profiles at 440 nm [26].

## Authors' contributions

AY participated in the design of the study and the writing of the manuscript, and carried out all of the experimental studies. RT participated in the design of the study, HPLC and physiological analyses and the writing of the manuscript. AT participated in the design of the study and the writing of the manuscript. All authors read and approved the final manuscript.
